# Cardiac Arrest as a Consequence of Air Embolism: A Case Report and Literature Review

**DOI:** 10.1155/2016/8236845

**Published:** 2016-11-27

**Authors:** Zia Ur Rahman, Ghulam Murtaza, Mohsin Pourmorteza, Wael K. El Minaoui, Pooja Sethi, Peyman Mamdouhi, Timir Paul

**Affiliations:** ^1^Department of Internal Medicine, East Tennessee State University, Johnson City, TN, USA; ^2^Blue Ridge Pulmonary Associates, Kingsport, TN, USA

## Abstract

Air embolism is an infrequent but potentially catastrophic complication. It could be a complication of invasive procedures including surgery, central line placement, positive pressure ventilation, trauma, hemodialysis, pacemaker placement, cardiac ablation, and decompression sickness. Usually, it does not cause any hemodynamic complication. In rare cases, it could lodge in the heart and cause cardiac arrest. We present a case of an 82-year-old white female who underwent computed tomography (CT) guided biopsy of right lung pulmonary nodule. When she was turned over after the lung biopsy, she became unresponsive and developed cardiopulmonary arrest. She underwent successful resuscitation and ultimately was intubated. CT chest was performed immediately after resuscitation which showed frothy air dense material in the left atrium and one of the right pulmonary veins suggesting a Broncho venous fistula with air embolism. Although very rare, air embolism could be catastrophic resulting in cardiac arrest. Supportive care including mechanical ventilation, vasopressors, volume resuscitation, and supplemental oxygen is the initial management. Patients with cardiac, neurological, or respiratory complications benefit from hyperbaric oxygen therapy.

## 1. Introduction

Although uncommon, air embolism is a life threatening complication. Surgery including laparoscopic surgery [1], vascular procedures such as peripheral vascular access, trauma, diving, and barotrauma from mechanical ventilation are the common causes of air embolism. We present a case of cardiac arrest from air embolism after the fine-needle lung biopsy.

## 2. Case Description

**Figure 1:**
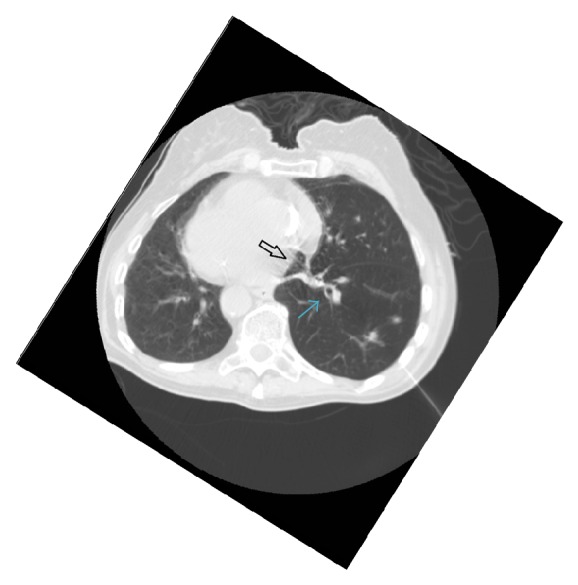
Arrow pointing towards collection of air in the left atrium (solid white arrow) and the right pulmonary veins (blue arrow) suggesting a Broncho venous fistula with air.

An 82-year-old white female with past medical history significant for chronic obstructive pulmonary disease atrial fibrillation, chronic hyponatremia, congestive heart failure, stroke, and hypertension who presented to the radiology department in our tertiary care center for computed tomography (CT) guided biopsy for further evaluation of recently diagnosed 1.9 cm right lower lobe pulmonary nodule and mediastinal lymphadenopathy highly suspicious for primary lung malignancy. A 19-gauge guiding needle was advanced to the vicinity of a small mass in the peripheral third of the mid right lower lobe of the lung. Three 22-gauge Chiba biopsy needles were sequentially placed through the guiding needle and cytologic material was aspirated and reviewed. Subsequently three 20-gauge core biopsy specimens were obtained from the mass. There was volume of hemorrhage in area of biopsy but no pneumothorax or other immediate complications were noted. When she was turned over after the lung biopsy, she became unresponsive and developed cardiopulmonary arrest. A code blue was called; cardiopulmonary resuscitation was performed following ACLS guidelines. She could regain spontaneous circulation with epinephrine. She was intubated for mechanical ventilation and admitted to the hospital in medical intensive care unit. CT chest was performed immediately after resuscitation which showed frothy air dense material in the left atrium and one of the right pulmonary veins suggesting a Broncho venous fistula with air embolism (Figure 1). CT head was obtained as well at the same time that showed no acute intracranial findings. Bedside transthoracic echo with contrast performed a few hours later was completely normal. There was no evidence of air bubble in atria or ventricles, ejection fraction was 55–60%, and right ventricle size and function were normal as well. Patient was placed in Trendelenburg position and was subsequently sent to hyperbaric oxygen chamber for treatment of air embolism. Patient tolerated the hyperbaric oxygen therapy very well. Patient was kept on the mechanical ventilator overnight. She was successfully weaned off from the mechanical ventilation and extubated on the next day of admission.

## 3. Pathophysiology

Air embolism results from entry of air into the vasculature and it could be categorized as arterial or venous based on the blood vessel involved. Arterial air embolism has worse prognosis as compared to venous air embolism as it could cause tissue ischemia when blood supply is halted because of lodgment of embolism in the arterioles and capillaries. Air embolism needs the presence of a pressure gradient favoring the passage of air into the vasculature and a direct communication between the source of air and blood vessels. Neurosurgery and ear, nose, and throat surgeries done in sitting position pose a higher risk for venous air embolism when compared to other surgeries, due to the presence of this pressure gradient [2, 3]. Venous air embolism causes injury through obstruction of blood flow from the right side of the heart to the left. This is due to mechanical obstruction of the right ventricular pulmonary outflow tract and pulmonary vasculature and to poor understanding of pulmonary vasoconstrictive mechanisms. Venous air embolism can result in considerable hypoxemia from ventilation-perfusion mismatch and shunt. With large emboli, systemic hypotension, myocardial ischemia, and arrhythmias can occur resulting in death [4]. In general, fatality of venous air embolism depends on the total volume of air entering the circulation, rate of entry, and destination. 300 to 500 mL of air introduced at a rate of 100 mL/sec can be acutely fatal for humans [5]. As an example, a 14-gauge catheter with a pressure gradient of only 5 cm H_2_O is usually sufficient to create this much flow rate [6].

## 4. History and Clinical Features

Clinical features depend upon the amount of air entering the circulation. Small amount of air entry into vasculature is common and usually causes no symptoms and is self-limiting. Patients with air embolism present variably based on the end organ involved. Shortness of breath, tachypnea, rales, wheezing, and respiratory failure could occur when pulmonary venous circulation is involved. Chest pain, shortness of breath, elevated JVD, hypotension, and shock-like picture should point towards cardiac air embolism. Altered mental status, dizziness, lightheadedness, and focal neurological deficits occur when brain is the end organ in case of arterial air embolism. Similarly, tissue ischemia of any organ could result from arterial embolism of the involved tissue. Large amount of air entry could be life threatening and usually characterized by acute-onset right-sided heart failure from cor pulmonale (air embolism of pulmonary vasculature), an acute sense of impending doom (brain arterial embolism), sudden-onset loss of consciousness (brain arterial embolism), hemodynamic collapse, or cardiac arrest (cardiac air embolism) [7].

## 5. Physical Examination

Signs of air embolism depend upon the end organ supplied by the involved vasculature. These include tachycardia, bradycardia, hypotension, a water-wheel or mill-wheel murmur (a characteristic splashing auscultatory sound due to the presence of gas in the cardiac chamber), shock-like picture, cardiac arrest, crackles, wheezing, tachypnea, hypoxemic respiratory failure altered mental status, focal neurological deficits, syncope, coma, crepitus in superficial vessels if skin is involved, and bubbles within the retinal arteries.

## 6. EKG, Imaging, and Laboratory Tests

EKG may show tachycardia and right heart strain pattern (peaked P wave, RBBB, and right axis deviation). Arterial air embolism could result in acute ischemia or infarction pattern on EKG. Chest X-ray could be normal or it may show pulmonary edema, pulmonary artery enlargement, and atelectasis or intracardiac air. Air present in the main pulmonary artery (although very rare) is pathognomonic of air embolism. ABG may indicate hypoxemic (more common) or hypercapnic respiratory failure. VQ scan could show VQ mismatch in cases of massive air embolism. The rapid resolution of the perfusion defects (within 24 hours on repeat VQ scan) may help differentiate venous air embolism and other forms of pulmonary thromboembolism [8]. CT chest may show air emboli in central veins, right ventricle, pulmonary artery, or heart. Echocardiography could sometime be used to rapidly identify air in the cardiac chambers or great veins, right ventricular dilatation, or pulmonary hypertension [9].

*Diagnosis.* When considering acute air embolism as a cause of acute patient demise, other causes of acute pulmonary, cardiac, or neurological decompensation (H's and T's) should be kept in mind and these should be ruled out by careful history, physical examination, and laboratory and imaging data. Air embolism should be considered in patients who develop sudden and acute cardiac, pulmonary, or neurological decompensation and who have obvious risk factors present for air embolism as described above. In such patients, presence of air in a particular organ on imaging studies should strongly suggest the diagnosis of air embolism. One needs to remember that no specific laboratory test, physical finding, or patient symptom may yield a timely diagnosis. Yet, air embolism could be acutely life threatening, so prompt recognition is imperative [10]. It is usually a clinical diagnosis based on high index of suspicion when other life threatening causes of acute decompensation are ruled out.

## 7. Treatment

A patient with venous thromboembolism should be immediately placed in left lateral decubitus position, Trendelenburg position, or left lateral decubitus head down position while a patient with arterial air embolism should be placed in supine position [11]. Treatment of acute air embolism depends upon the clinical condition of the patient. In most patients, therapy is supportive and includes airway support, high flow oxygen, volume resuscitation, vasopressors, ACLS, and mechanical ventilation. Patients who develop seizures should be treated with standard medical therapy for seizures. Hemodynamically unstable patients and patients with end organ damage or neurological deficits should be treated with definitive therapy which includes hyperbaric oxygen [12], withdrawal of air from the right atrium, or cardiac massage [13]. Hyperbaric oxygen therapy plays a major role in successful resuscitation of these patients [14]. When air embolism is suspected, placement of the patient in the left lateral decubitus position, initiating closed chest massage, or, if possible, aspiration of air through a right atrial or Swan-Ganz catheter are all acceptable forms of treatment. The patient should also be given 100% oxygen [4].

## 8. Discussion

CT guided lung biopsy is a commonly performed procedure in most hospitals to diagnose various pulmonary conditions. The occurrence of air embolism complicating CT guided lung biopsies is very rare. A study conducted in Japan including 9783 patients who underwent CT guided lung biopsies reported 0.061% incidence of air embolism [15]. The reported incidence of air embolism after CT guided transthoracic lung biopsy that ranges from 0.02% to 0.06% [15, 16], but failure to diagnose in timely manner can have grave consequences [17]. Cardiac arrest because of air embolism is an extremely rare but life threatening complication of CT guided transthoracic lung biopsies. Few cases of fatal cardiac arrest complicating transthoracic lung biopsy were reported [18–21]. A recent large multicenter case control study done in Japan looked at the risk factors for the development of systemic air embolism after CT guided lung biopsies. They concluded that parenchymal hemorrhage during the procedure, lesions in the lower lobe, and the use of larger biopsy needles may be risk factors for systemic air embolism by percutaneous CT guided lung biopsy [22]. One study looked at the complications of 1010 cases of CT guided lung biopsies performed in one institution and four cases of nonfatal air embolism were identified [16]. One case of nonfatal air embolism was reported in another case report [23].

In our case, patient was sedated and did not cough during the procedure. Pulmonary nodule being biopsied was solid without any cystic or cavitary features. Radiologist used the coaxial technique and size of the needle used was relatively large that we believe might have contributed to this complication. At our center, CT guided lung biopsies are performed by an experienced radiologist and, on an average, there are 2-3 cases per week. Coaxial method is commonly used now to reduce risk of pneumothorax that is one of the commonest complications of this procedure. Parenchymal hemorrhage and pneumothorax have occurred in the past, but air embolism to an extent causing cardiac arrest has never occurred before and this is the first reported case of such complication at our institution. Our case stresses the importance of being aware that systemic air embolism can happen as very rare but dangerous complication and we should have facilities to readily provide urgent treatment; otherwise it could be fatal.

## 9. Conclusion

When caring for critically ill patients, nursing staff and physicians should be aware of the etiology, clinical features, and immediate treatment of potentially lethal air embolism. Air embolism should be a differential in certain cardiac arrest patients when there is sufficient clinical suspicion especially after a surgical procedure or manipulation of blood vessels by any means. Due to its life threatening nature, early identification and treatment of this condition require prudent clinical judgement in a given clinical setting. One should be familiar with the clinical setting where air embolism occurs, as prevention is the best treatment.
